# The Effectiveness and Safety of Direct-Acting Antivirals in the Treatment of Hepatitis C Virus in Saudi Arabia: A Nationwide Study Based on the Saudi Ministry of Health Surveillance Data From 2017 to 2021

**DOI:** 10.7759/cureus.42780

**Published:** 2023-08-01

**Authors:** Tasneem S. AL-Ahmari, Adel F Alotaibi, Areej I Aljasser, Abdulrahman I Aljasser, Anwar M Eldaw, Eman E Abd-Ellatif

**Affiliations:** 1 Public Health and Preventive Medicine, Ministry of Health, Riyadh, SAU; 2 Epidemiology, Assistance Agency for Preventive Health, Ministry of Health, Riyadh, SAU; 3 Uorology, Huraymala General Hospital, Ministry of Health, Riyadh, SAU; 4 Infectious Disease/Public Health and Preventive Medicine, Ministry of Health, Riyadh, SAU; 5 Public Health and Preventive Medicine, Faculty of Medicine, Mansoura University, Mansoura, EGY

**Keywords:** ministry of health (moh), effectiveness and safety, direct-acting antivirals, kingdom of saudi arabia (ksa), hepatitis c virus (hcv)

## Abstract

Background and objective

While the Kingdom of Saudi Arabia (KSA) has had a hepatitis C virus (HCV) elimination program in place since 2015, there have been limited studies investigating the effectiveness and safety of direct-acting antivirals (DAAS) based on the Ministry of Health (MOH) surveillance data. In light of this, this study was conducted to assess the effectiveness and safety of DAAS (glecaprevir/pibrentasvir, daclatasvir/sofosbuvir, or other combinations) in treating HCV cases in Saudi Arabia as per the MOH data from 2017 to 2021.

Methods

This was a retrospective cohort study involving recorded HCV cases in the national hepatitis surveillance database of MOH across all regions of KSA from 2017 to 2021. Statistical analyses were performed using IBM SPSS Statistics software (IBM Corp., Armonk, NY). Continuous variables were expressed as mean ± standard deviation (SD), and categorical variables were presented as numbers (percentages). An independent t-test was used for continuous variables, and a Chi-square analysis was used for categorical variables. A confidence interval of 95%, a margin of error of 0.05, a precision of 2%, and a 5% level of significance were employed.

Results

Regarding demographic characteristics, age was significantly associated with HCV infection (p=0.002). Similarly, nationality had a highly significant association with HCV infection (p=0.004). Regarding clinical characteristics, creatinine levels were significantly associated with HCV infection (p=0.009). As for effectiveness, all participants had a positive polymerase chain reaction* *(PCR) for HCV at enrolment (n=4806) and were DAAS-naïve. After the completion of the first DAAS course, 99.5% (4781) had negative PCRs 12 weeks post-treatment completion; however, the PCR remained positive for some patients (0.5%, n=25), which became negative after receiving the second DAAS course, resulting in complete cure of HCV infection and a 100% negative PCR among all participants. With regard to safety, no side effects were recorded in the cohort and hence the safety aspect was not analyzed.

Conclusion

Univariate analysis revealed that nationality (non-Saudi), age, and creatinine levels were significantly associated with HCV infection. However, only nationality showed a significant association with HCV infection following multivariate logistic regression adjustment. We believe that these insights will help guide the creation of clinical guidelines and promote evidence-based decision-making in the management of HCV in Saudi Arabia.

## Introduction

The hepatitis C virus (HCV) infection is a blood-borne viral infection that leads to either acute (approximately 30%) or chronic (70%) infection. Unfortunately, unlike for hepatitis B virus (HBV), no vaccination to prevent HCV is currently available. The Eastern Mediterranean Region (EMR) has been reported to have the highest prevalence of viral hepatitis C globally, accounting for 2.3% of cases [1]. The HCV prevalence in the Kingdom of Saudi Arabia (KSA) as reported by the Ministry of Health (MOH) in 2022 was 0.23% [2]. KSA has had an HCV elimination program in place since 2015 [3]. HCV prevalence increases with increasing age, from approximately 4.5% in children to 8.6% in young adults and approximately 12% in adults aged above 55 years [3]. HCV prevalence among drug users in Saudi Arabia is 69% and is believed to increase in the future along with increasing cases of complicated liver cirrhosis [3].

In EMR, HCV infection is the most serious form of hepatitis as it is the most common cause of liver transplantations among the adult population, and, in conjunction with HBV, 7% of cirrhosis and liver cancer cases have been attributed to it [4]. HCV burden of undiagnosed cases is high as 80% in KSA [5]. Of note, 15% of HCV cases are diagnosed through mandatory pre-marital screening [5]. The median age of Saudi patients with HCV cases is 60 years [6]. Individuals aged above 15 years have a 45 times higher annual incidence of HCV compared to those aged below 15 years. The 10-year risk of liver cirrhosis depends on the severity of hepatitis, i.e., mild (10%), moderate (44%), and severe (100%) [7]. While the five-year survival rate for patients with compensated cirrhosis is as high as 90%, it is 50% for those with decompensated cirrhosis [7]. The annual risk of decompensation among patients with compensated cirrhosis is 3.9% [7]. HCV cases with cirrhosis have a 0-3% risk of developing hepatocellular carcinoma (HCC) [8]. The national HCV policy for the use of direct-acting antivirals (DAAS) was established in the year 2015 by the Ministry of National Guard Health Affairs in Saudi Arabia [9]. However, MOH is leading the elimination project, by implementing treatment guidelines, protocols, and screening policy, as well as control and supervision of the process and outcomes. As for the criteria to initiate DAAS therapy, the Saudi Association for the Study of Liver Diseases and Transplantation (SASLT) recommends that All HCV cases detected in KSA are treated with DAAS therapy regardless of HCV therapy history [10].

According to the 2019 American Association for the Study of Liver Diseases (AASLD) guidelines, all HCV cases aged three years and above are eligible for DAAS therapy [11]. The proportion of HCV-infected individuals treated with DAAS in KSA is 2.5% annually. While there is a lack of studies in the literature on the total HCV healthcare expenditure, Saudi Arabia reportedly spent 16.5 billion dollars in 2015 [12]. In 2014, there were 198 liver transplants, which cost approximately 18 million dollars, and 45% of these cases were HCV-related [12]. The national HCV screening program was launched in KSA in 2018 with the aim to screen all individuals aged above 40 years at least once in their lifetime. The goal of HCV therapy is to eradicate the infection altogether, while that of HBV therapy is to improve quality of life and prevent disease progression, transmission, or reactivation. It has been forecast that in the coming 20 years, 200,000 Saudi HCV patients will develop hepatic cirrhosis, and 1,500 of those patients will develop HCC annually [12]. The world health organization (WHO) plans to eliminate viral hepatitis by 2030, and the program involves reducing 90% of new chronic infections and a reduction in mortality by 65%. Second-generation DAAS are expected to play a critical role in eradicating HCV in the future [12]. Nonetheless, Saudi Arabia expects to achieve the target of diagnosing 90% of HCV infections and treating 80% of HCV cases by 2048. Additionally, KSA aims to achieve the goal of HCV elimination by 2051. DAAS are 94% more cost-effective compared to other HCV treatment options and are expected to reduce direct health costs for HCV treatment by 30%. It has been suggested that the worldwide use of DAAS would lead to an estimated reduction in decompensated cirrhosis (61%), HCC (45%), liver transplants (50%), and liver-related deaths (61%) [12].

## Materials and methods

Study design and population

We employed a retrospective cohort design for this study. It was intended to involve all identified HCV patients who received ("exposed") or did not receive ("non-exposed") DAAS therapy (glecaprevir/pibrentasvir, daclatasvir/sofosbuvir, or other combinations) as documented in the MOH hepatitis program database, including all nationalities and genders aged three years or above as reported by all MOH hospitals in all regions of KSA, regardless of whether they had been treated with other non-DAAS therapies or not, and irrespective of the presence of comorbidities (cirrhosis or renal failure) or coinfections such as HBV or HIV infections.

Inclusion criteria

The inclusion criteria were polymerase chain reaction (PCR)-confirmed HCV cases that are registered in the MOH hepatitis program database.

Exclusion criteria

The exclusion criteria were as follows: non-HCV hepatitis cases; any hepatitis case not registered in the Saudi MOH hepatitis program database; any HCV case reported by non-MOH hospitals; HCV cases that were lost to follow-up; and HCV cases aged below three years.

Study setting and data source

The data were record-based and retrieved from the electronic hepatitis surveillance system, a comprehensive and flexible online-based platform that facilitates routine nationwide data collection.

Sample size

Everyone who participated in the national (MOH) hepatitis surveillance system (n=4806) was included.

Statistical analysis

Continuous variables were expressed as mean ± standard deviation (SD), and categorical variables were presented as numbers (percentages). An independent t-test was used for continuous variables, and a Chi-square test was used for categorical variables. A confidence interval of 95%, a margin of error of 0.05, a precision of 2%, and a 5% level of significance were employed. Statistical analyses were performed using IBM SPSS Statistics software (IBM Corp., Armonk, NY). Data transformation was not used. Missing data were dealt with by nullifying the missing values. Initial frequencies were calculated, followed by recoding the data and calculating clean frequencies; means were compared and cross-tabulations were measured.

Variables measured in HCV cases

The variables were as follows: age (above or below 50 years), gender (male, female), region (categorical), nationality (Saudi, non-Saudi), body mass index (BMI) (scale), genotype (categorical), comorbidities (yes/no), renal failure (yes/no), decompensation (yes/no), fibrosis scan (categorical), on dialysis (yes/no), creatinine level (scale), treatment duration (scale) and treatment type (categorical), and PCR (positive/negative).

Outcomes measures

Effectiveness

The primary endpoint was negative PCR 12 weeks post-treatment completion. Failure included drug cessation due to reported side effects or positive PCR 12 weeks post-treatment completion.

Safety

The primary endpoint was the rate and proportion of treatment adverse events (TAEs) including treatment discontinuation, death due to TAEs, emergency visits, and hospitalization due to TAE. 

DAAS therapy regimen and patient assignment

These were based on MOH protocol and depended on whether the patient had previously been exposed to DAAS therapy or not, if the patient was taking other drugs that interact with DAAS therapy, and for females, whether pregnant or not. Class 1 DAAS assigned included daclatasvir (400 mg)/sofosbuvir (60 mg) for 12 or 24 weeks and class 2 DAAS involved glecaprevir (300 mg)/pibrentasvir (120 mg) for 8-24 weeks. The indications to assign patients to either class 1 or class 2 of DAAS were as follows: if the patient was newly infected, had no other liver problems or complications, had never been treated with DAAS, and there was no other reason to be given class 2 DAAS, then the patient was given class 1 DAAS. However, if the treating consultant had a justified reason for assigning a patient to class 2 DAAS, they were expected to report this reason to the MOH Department of the National Program to Combat HCV for approval and supply [13].

## Results

Demographic and clinical characteristics

Table 1 shows the demographic and clinical characteristics of the participants, including age, creatinine (mg/dl), gender, nationality, region, comorbidities [diabetes mellitus (DM), hypertension (HTN), cardiovascular disease (CVD), etc.], HBV coinfection, HIV coinfection, decompensation (i.e., with ascites, encephalopathy and/or varicella), dialysis, genotypes, FibroScan (liver fibrosis staging), and treatment regimens.

**Table 1:** Participant characteristics ^a^Independent t-test. ^b^Chi-square-test. ^c^Fisher's exact test. *Significant at p<0.05. **Significant at p<0.01 PCR: polymerase chain reaction; BMI: body mass index; HBV: hepatitis B virus; HIV: human immunodeficiency virus; SD: standard deviation

Variable		Negative PCR	Positive PCR	P-value
		N=4863	N=25	
Age^a^, years	Range	5–99	16–84	0.002**
	Mean ± SD	50.02 ± 16.18	60.38 ± 17.07	
Creatinine^a^, mg/dl	Range	0.0034–6.93	0.0045–5.44	0.009**
	Mean ± SD	0.537 ± 0.953	1.07 ± 1.67	
BMI^a^, kg/m^2^	Range	11.4–64.9	21.4–31.2	0.067
	Mean ± SD	26.9 ± 5.36	24.8 ± 3.02	
Gender^b^, n (%)	Male	2191 (99.3%)	16 (0.7%)	0.272
	Female	1731 (99.5%)	9 (0.5%)	
Nationality^b^, n (%)	Saudi	3163 (99.6%)	14 (0.4%)	0.004**
	Non-Saudi	753 (98.6%)	11 (1.4%)	
Region^b^,n (%)	West	1451 (99.4%)	9 (0.6%)	0.681
	East	611 (99.7%)	2 (0.3%)	
	Central	910 (99.7%)	3 (0.3%)	
	Other regions	1883 (99.4%)	11 (0.6%)	
Comorbidities^b^,n (%)	No	2451 (99.6%)	11 (0.4%)	0.092
	Yes	1209 (99.1%)	11 (0.9%)	
HBV coinfection^c^,n (%)	No	3838 (99.4%)	25 (0.6%)	0.919
	Yes	13 (100%)	0 (0%)	
HIV coinfection^c^,n (%)	No	3849 (99.4%)	25 (0.6%)	0.987
	Yes	2 (100%)	0 (0%)	
Decompensation^c^,n (%)	No	3774 (99.4%)	24 (0.6%)	0.399
	Yes	77 (98.7%)	1 (1.3%)	
Dialysis^c^,n (%)	No	3270 (99.3%)	22 (0.7%)	0.467
	Yes	581 (99.5%)	3 (0.5%)	
Genotypes^b^,n (%)	GT-4	1538 (99.2%)	12 (0.8%)	0.9
	GT-3	520 (99.0%)	5 (1%)	
	GT-1	964 (99.4%)	6 (0.6%)	
	Other (2, 5, 6, 7)	216 (99.1%)	2 (0.9%)	
FibroScan^b^,n (%)	F4	1005 (99.4%)	6 (0.6%)	0.155
	F3	541 (99.3%)	4 (0.7%)	
	F2	646 (98.8%)	8 (1.2%)	
	F1	1061 (99.3%)	7 (0.7%)	
	F0	510 (100%)	0 (0%)	
Regimens^b^,n (%)	Glecaprevir/pibrentasvir	1081 (100%)	0 (0%)	0.023*
	Daclatasvir/sofosbuvir	1922 (99.4%)	12 (0.6%)	
	Other combinations	1753 (99.3%)	13 (0.7%)	

Our findings showed that age was significantly associated with HCV infection (p=0.002). The mean age of the PCR-positive group (60.38 ± 17.07 years) was higher than that of the PCR-negative group (50.02 ± 16.18 years). Similarly, nationality showed a highly significant association with HCV infection (p=0.004). Among Saudi participants, 99.6% tested negative for HCV, while only 0.4% tested positive for HCV. On the other hand, among non-Saudi participants, 98.6% tested negative for HCV, and 1.4% tested positive. These results indicate that the likelihood of a positive HCV test result differs between Saudis and non-Saudis. Conversely, gender and region did not show a significant association with HCV infection (p>0.05).

Regarding clinical characteristics, creatinine levels were significantly associated with HCV infection (p=0.009). It was higher in the PCR-positive group (1.07 ± 1.67 mg/dl) compared to the PCR-negative group (0.537 ± 0.953 mg/dl). Likewise, the results showed a significant association between the choice of treatment regimen and HCV infection (p=0.023). Specifically, the glecaprevir/pibrentasvir regimen demonstrated a significantly lower likelihood of HCV infection than the other two regimens. Hence, it can be assumed that the glecaprevir/pibrentasvir regimen is more successful in treating HCV cases than other regimens. On the contrary, no statistically significant association was found between HCV infection and BMI, comorbidities, HBV coinfection, HIV coinfection, decompensation, dialysis, genotype, and FibroScan (p>0.05).

Predictors and response

Table 2 shows the association of predictors with HCV infection. Simple and multiple logistic regression analyses were used to calculate the crude and adjusted odd ratios, respectively. Variables of nationality, comorbidities, dialysis, age in years, BMI, and creatinine levels were assessed using a multivariate logistic regression model.

**Table 2:** Crude and adjusted OR (95% CI) based on logistic regression analysis identifying the association between study variables and HCV infection *Significant at <0.05. **Significant at <0.01 Crude odds ratios measure the association between the two variables without controlling for other variables, detected by simple logistic regression. Adjusted odds ratios measure the association between the two variables while controlling for other variables in the model, detected by multiple logistic regression OR: odds ratio; CI: confidence interval; BMI: body mass index; NA: not applicable

Variable	Categories	Crude odds ratio		Adjusted odds ratio	
		OR (95% CI)	P-value	OR (95% CI)	P-value
Gender	Male	1.405 (0.619–3.186)	0.416	NA	
	Female (reference)				
Nationality	Saudi	0.303 (0.137–0.670)**	0.003	0.062 (0.007–0.546)*	0.012
	Non-Saudi (reference)				
Region	West	1.062 (0.439–2.569)	0.894	NA	
	East	0.560 (0.124–2.535)	0.452	NA	
	Central	0.564 (0.157–2.028)	0.381	NA	
	Other cities (reference)				
Comorbidities	No (reference)				
	Yes	2.027 (0.876–4.689)	0.099	1.972 (0.279–13.957)	0.497
Decompensation	No (reference)				
	Yes	2.042 (0.273–15.288)	0.487	NA	
Dialysis	No (reference)				
	Yes	0.767 (0.229–2.572)	0.668	0.344 (0.028–4.230)	0.404
Genotype	Others (reference)				
	GT-1	0.672 (0.135–3.353)	0.628	NA	
	GT-3	1.038 (0.200–5.394)	0.964	NA	
	GT-4	0.843 (0.187–3.791)	0.823	NA	
Age		1.042 (1.015–1.069)**	0.002	1.039 (0.974–1.108)	0.247
BMI		0.911 (0.786–1.056)	0.215	0.971 (0.803–1.175)	0.764
Creatinine		1.353 (1.037–1.767)*	0.026	1.316 (0.672–2.579)	0.423

Regarding gender, the chance of HCV infection among males was 1.405 times higher than in females. However, this association was not statistically significant (OR: 1.405, 95% CI: 0.619-3.186). As for nationality, Saudi nationality had a significantly lower association with HCV infection. The chance of HCV infection among Saudi nationals compared to non-Saudi individuals was 0.303 (OR: 0.303, 95% CI: 0.137-0.670) with a p-value of 0.003, indicating a lower odds of HCV infection among Saudi nationals. Also, after adjusting for other variables, the odds ratio was 0.062 (95% CI: 0.007-0.546), indicating significantly lower odds of HCV infection among Saudi individuals compared to non-Saudi individuals (p=0.012). Regarding region, no significant association was found between HCV infection and regions (West, East, Central, and others). The crude odds ratios were not statistically significant, indicating that the region that individuals hailed from did not significantly impact HCV infection risk (p>0.05).

In terms of comorbidities, it was found that HCV infection was higher in individuals with comorbidities compared to those without comorbidities. However, this association was not statistically significant (OR: 2.027, 95% CI: 0.876-4.689, p=0.099). Additionally, after adjusting for other variables, the odds ratio was 1.972 (95% CI: 0.279-13.957), still not reaching statistical significance (p=0.497). As for decompensation, no significant association was detected with HCV infection. The chance of HCV infection for decompensation compared to no decompensation was 2.042 (95% CI: 0.273-15.288); this association was not statistically significant (p=0.487).

With respect to dialysis, no statistically significant association was found with HCV infection. The crude odds ratio for dialysis compared to no dialysis was 0.767 (95% CI: 0.229-2.572), indicating no substantial difference in HCV infection odds. After adjusting for other factors, the odds ratio was 0.344 (95% CI: 0.028-4.230), with no statistical significance (p=0.404). With regard to genotypes, no significant association was found between different genotypes (GT-1, GT-3, GT-4, and others) and HCV infection. The crude odds ratios were not statistically significant, suggesting that genotype did not significantly impact HCV infection risk (p>0.05). As for age, with an increase of one year in age, the chance of HCV infection increased multiplicatively by 1.042. This association was statistically significant (OR: 1.042, 95% CI: 1.015-1.069, p=0.002). However, after adjusting for other variables, age no longer showed a significant association with HCV infection (OR: 1.039, 95% CI: 0.974-1.108, p=0.247).

Regarding BMI, there was no significant association with HCV infection (p>0.05). With each unit increase in the BMI, the chance of HCV infection increased multiplicatively by 0.911 and 0.971 in crude and adjusted odd ratios, respectively. Finally, with regard to creatinine levels, each unit increase in the creatinine level increased the chance of HCV infection by 1.353 times. This association was statistically significant (OR: 1.353, 95% CI: 1.037-1.767, p=0.026). However, after adjusting for other variables, creatinine level showed a non-significant association with HCV infection (OR: 1.316, 95% CI: 0.672-2.579, p=0.423).

Univariate analysis revealed that nationality (non-Saudi), age, and creatinine levels were significantly associated with HCV infection. However, only nationality showed a significant association with HCV infection after multivariate logistic regression adjustment. These findings demonstrate the importance of incorporating these parameters when evaluating the risk and prevalence of HCV infection in the Saudi population.

DAAS treatment course

Assigning patients to DAAS therapy was done according to the MOH hepatitis protocol. The first DAAS regimens received were as follows: glecaprevir/pibrentasvir: 22.1% (n=1081), daclatasvir/sofosbuvir: 39.6% (n=1934), and other combinations: 36.1% (n=1766). The second DAAS regimens used were as follows: glecaprevir/pibrentasvir: 4% (n=1), daclatasvir/sofosbuvir: 16% (n=4), and other combinations: 80% (n=20) (Table 3). The mean therapy duration was 12 weeks.

**Table 3:** Distribution of first DAAS versus second DAAS regimens in the study DAAS: direct-acting antivirals

Regimens	First DAAS	Second DAAS
	N=4781, n (%)	N=25, n (%)
Glecaprevir/pibrentasvir	1081 (22.6%)	1 (4.0%)
Daclatasvir/sofosbuvir	1934 (40.5%)	4 (16.0%)
Other combinations	1766 (36.9%)	20 (80.0%)

Association between regimens and genotypes

Table 4 shows the distribution of different regimens across various genotypes. A Chi-square test was used to detect the association between these regimens and genotypes. The statistical analysis showed a highly significant association between the regimens and different genotypes (p<0.001). Notably, glecaprevir/pibrentasvir was predominantly effective in treating genotype-3, which accounted for 59.7% of the cases within this regimen. The low representation of genotype-1 in this regimen (1.5%) was also noteworthy. In contrast, daclatasvir/sofosbuvir had a more balanced distribution across genotypes. It was most effective for genotype-4 at 44.1%, followed by genotype-1 and genotype-3, accounting for 25.4% and 21.5%, respectively, and less effective for other genotypes (9.0%). For other combinations, genotype-4 accounted for the majority at 51.5%, followed by genotype-1 (34.4%), genotype-3 (10%), and other genotypes (4.0%).

**Table 4:** Association between first DAAS regimens and genotypes *Significant at <0.01 The p-value was calculated using the Chi-square test DAAS: direct-acting antivirals

Regimens	Genotype-1	Genotype-3	Genotype-4	Other genotypes	Total	P-value
	N=970, n (%)	N=525, n (%)	N=1546, n (%)	N=218, n (%)	N=3259, n (%)	
Glecaprevir/pibrentasvir	1 (1.5%)	40 (59.7%)	8 (11.9%)	18 (26.9%)	67 (100%)	<0.001*
Daclatasvir/sofosbuvir	365 (25.4%)	309 (21.5%)	633 (44.1%)	129 (9.0%)	1436 (100%)	
Other combinations	604 (34.4%)	176 (10%)	905 (51.5%)	71 (4.0%)	1756 (100%)	
Total	970 (29.8%)	525 (16.1%)	1546 (47.4%)	218 (6.7%)	3259 (100%)	

Table 5 shows the association between regimens and genotypes for the HCV PCR-positive group. A highly statistically significant association was detected (p<0.001). Firstly, the glecaprevir/pibrentasvir regimen effectively treated one case in the genotype-4 group. Secondly, the daclatasvir/sofosbuvir regimen had a more diverse distribution across genotypes. One case (25.0%) fell under genotype-1, two cases (50.0%) under genotype-4, and one case (25.0%) under other genotypes. Finally, in the category of other combinations, there was also a varied distribution across genotypes. Genotype-4 had the highest representation at 45%, followed by genotype-1 and genotype-3 at 25% each, and other genotypes at 5%.

**Table 5:** Association between second DAAS regimens and genotypes for the HCV PCR-positive group *Significant at <0.01 The p-value was calculated using the Chi-square test DAAS: direct-acting antivirals; HCV: hepatitis C virus; PCR: polymerase chain reaction

Regimens	Genotype-1	Genotype-3	Genotype-4	Other genotypes	Total	P-value
	N=6, n (%)	N=5, n (%)	N=12, n (%)	N=2, n (%)	N=25, n (%)	
Glecaprevir/pibrentasvir	0 (0%)	0 (0%)	1 (100.0%)	0 (0%)	1 (100%)	<0.001*
Daclatasvir/sofosbuvir	1 (25.0%)	0 (0%)	2 (50.0%)	1 (25.0%)	4 (100%)	
Other combinations	5 (25.0%)	5 (25.0%)	9 (45%)	1 (5.0%)	20 (100%)	
Total	6 (24.0%)	5 (20.0%)	12 (48.0%)	2 (8.0%)	25 (100%)	

Association between genotypes and nationality

Table 6 shows the association between genotypes and nationality. A Chi-square test was used to detect this type of association. It revealed a highly statistically significant association between nationality and different genotypes. In the non-Saudi group, genotype-4 was the most prevalent one, accounting for 44.5% of cases, followed by genotype-1 at 27.7% and genotype-3 at 22.8%. Other genotypes represented a small portion (4.9%). In the Saudi group, a similar pattern was observed; genotype-4 was the most common one, representing 47.1% of cases. Genotype-1 accounted for 30.5%, and genotype-3 accounted for 14.3%. Other genotypes made up 8.2% of cases in the Saudi group. Also, it is interesting to note that genotype-3 had a higher representation in the non-Saudi group (22.8%) compared to the Saudi group (14.3%). This finding might indicate that genotype-3 is relatively more prevalent among non-Saudis. The results also revealed that other genotypes (i.e., GT-2, 5, and 6) have a relatively higher representation in the Saudi group than in the non-Saudi group.

**Table 6:** Association between genotypes and nationality *Significant at <0.01 The p-value was calculated using the Chi-square test

Genotypes	Non-Saudi	Saudi	Total	P-value
	N=429, n (%)	N=1995, n (%)	N=2424, n (%)	
Genotype-1	119 (27.7%)	608 (30.5%)	727 (30.0%)	<0.001*
Genotype-3	98 (22.8%)	285 (14.3%)	383 (15.8%)	
Genotype-4	191 (44.5%)	939 (47.1%)	1130 (46.6%)	
Other genotypes	21 (4.9%)	163 (8.2%)	184 (7.6%)	

As shown in Tables 4-5, treatment choices for HCV vary depending on the genotype of the virus. These findings highlight the significance of personalized medicine in effectively managing the disease. The observed differences in treatment selection underscore the importance of adopting genotype-specific approaches to achieve optimal outcomes for patients with hepatitis C. Additionally, the data shown in Table 6 reveal some interesting patterns in the distribution of hepatitis C genotypes among Saudi and non-Saudi individuals. Notably, genotype-4 showed a significant association with Saudi patients. These findings can be useful for understanding the epidemiology of hepatitis C and developing targeted strategies for prevention, diagnosis, and treatment based on the specific genotypes prevalent within different populations.

Safety

It is important to acknowledge that this study did not conduct a thorough analysis with regard to safety due to the absence of recorded data. Hence, it is crucial to avoid any bias favoring the effectiveness of DAAS by recognizing the limitation in safety evaluation. Future studies should strive to incorporate safety-related data to comprehensively assess the benefits and potential risks associated with the different treatment regimens used for managing hepatitis C. An inclusive analysis involving both efficacy and safety outcomes would provide a more balanced and comprehensive understanding of the treatment landscape for this condition.

## Discussion

The advent of DAAS therapy has ushered in a remarkable transformation in the treatment landscape of HCV. These ground-breaking medications have revolutionized HCV therapy in terms of effectiveness and safety profiles. This study aimed to provide an overview of the effectiveness and safety of DAAS in the treatment of HCV in Saudi Arabia based on a nationwide database spanning the period from 2017 to 2021. Overall, this study provides valuable insights into HCV infection demographics and clinical characteristics. The findings indicated that age and nationality are significant risk factors for HCV infection while creatinine levels and DAAS regimen choice are also associated with HCV infection. On the other hand, no significant associations were found between HCV infection and other clinical characteristics such as BMI, comorbidities, coinfection with hepatitis HBV or HIV, decompensation, dialysis, genotype, and liver fibrosis staging (FibroScan). Moreover, the current study found no significant association between HCV infection and patient gender and region.

Our primary and secondary objectives were to measure the effectiveness and safety of DAAS (glecaprevir/pibrentasvir, daclatasvir/sofosbuvir, or other combinations) in the treatment of HCV cases as recorded in the national hepatitis surveillance database of the MOH across all regions of KSA, from 2017 to 2021. Effectiveness was measured by recording HCV-RNA viral load using PCR at baseline and comparing it with that at 12 weeks post-treatment completion. Age was significantly associated with HCV infection, with the PCR-positive group having a higher mean age than the PCR-negative group. These findings are consistent with previous studies that also identified age as a risk factor for HCV infection [3]. Also, this study revealed a significant association between creatinine levels and HCV infection, with higher creatinine levels observed in the PCR-positive group. This finding suggests a potential link between renal function and HCV infection, warranting further investigation to understand the underlying mechanism [14]. Furthermore, nationality was also found to have a highly significant association with HCV infection, indicating a disparity in the likelihood of testing positive for HCV between Saudis and non-Saudis. Similar trends have been observed in previous international studies [15]. Saudi nationality was associated with a lower odds ratio for HCV infection than non-Saudi individuals, indicating a lower risk among Saudi nationals. This association remained significant even after adjusting for other variables, highlighting the importance of considering nationality when evaluating HCV infection risk in the Saudi population. Our interpretation of the findings mentioned above is that nationality might be a proxy indicator for these genotyping differences and, accordingly, variation in response to DAAS therapy. It is important to consider the HCV genotype distribution in Saudi Arabia, with genotype-4 (GT-4) being the most prevalent in the region.

The effectiveness of DAAS may vary depending on the HCV genotype. Glecaprevir/pibrentasvir was effective in managing genotype-3 cases, which accounted for 59.7% of the instances within this regimen. Conversely, the representation of genotype-1 in this regimen was relatively meager, comprising only 1.5% of the cases. This marked distribution discrepancy suggested that glecaprevir/pibrentasvir may exhibit heightened efficacy for genotype-3 patients while it is less frequently employed for genotype-1 individuals. In contrast, the glecaprevir/pibrentasvir regimen appeared to exhibit a more balanced pattern across genotypes. It demonstrated the greatest effectiveness in combating genotype-4, constituting 44.1% of the cases. Furthermore, this regimen also manifested effectiveness against genotype-1 (25.4%) and genotype-3 (21.5%), albeit to a lesser extent against other genotypes (9.0%). For the remaining combinations of regimens, genotype-4 represented the majority at 51.5%, followed by genotype-1 (34.4%), genotype-3 (10%), and other genotypes (4.0%). Generally, genotype-4 was the most treated genotype across all regimens. These findings are consistent with several previous studies that emphasize the varying effectiveness of distinct regimens concerning different genotypes [16]. Collectively, the results underscore the utmost significance of tailoring treatments according to genotype for achieving optimal therapeutic outcomes in HCV patients. These findings have noteworthy implications in terms of selecting treatments and implementing personalized medicine approaches. They offer invaluable insights into the effectiveness of specific regimens depending on the patient's genotype.

Our second objective was to measure DAAS safety, i.e., the rate and proportion of TAEs including treatment discontinuation, death due to TAEs, emergency visits, and hospitalization due to TAEs; however, unfortunately, there was a dearth of data regarding side effects, and hence analysis for DAAS safety was not conducted. Nonetheless, several studies worldwide have found that DAAS are well-tolerable and highly effective [17]. However, one single-center study found that around 3% of Saudi HCV cases who received DAAS reported experiencing some minor side effects like diarrhea and vomiting [1]. So far, no HCV cases have reportedly discontinued any DAAS treatment due to severe drug adverse events either globally or in KSA [14]. In our study, no side effects were reported; this could be due to poor recording or missing data. 

Recommendations

We recommend that steps be taken to enhance the MOH surveillance data recording regarding side effects experienced by patients and laboratory results, especially in terms of parameters such as albumin, bilirubin, alanine transaminase (ALT), prothrombin, platelets, and hemoglobin. We also believe that it is important to further analyze the safety of DAAS therapy among HCV patients across all MOH hospitals in KSA. Further studies are needed to investigate whether non-interferon regimens are the best modality for both HCV-HBV coinfections as it is yet unclear what the optimal regimen is. And, we also recommend further investigating reactivation due to resistance in patients with hepatitis C and B coinfection. It is vital to continue research efforts to further investigate the effectiveness and safety of DAAS by creating comprehensive clinical pharmacological data and drug interaction profiles for DAAS. Further studies should also be conducted on the impact of DAAS therapy on the natural history and development of HCC. 

Limitations

Since the data we used were secondary, we could not report the response rate or comment on non-response bias or dropout rate, or assess the internal consistency of the data collection form that belongs to the MOH hepatitis surveillance system. In general, PCR was used by MOH as an alternative test to measuring viral load. After 2018, no genotyping or FibroScan was done due to the change in MOH policy and protocol; hence, a big portion of data was considered missing and nullified and analysis was conducted based on year 2017 and 2018 data only. This study originally intended to compare the effectiveness of DAAS therapy among hepatitis C patients who were DAAS-naïve versus those with DAAS exposure history; however, based on data findings, all participants were found to be DAAS-naïve. Hence, we modified our approach and considered those who failed DAAS therapy in the first instance and received a second DAAS course as "exposed to DAAS" and then compared them to patients who received the DAAS course once and got cured ("DAAS-naïve"). Also, the study was confined to Saudi residents alone and hence the findings may not be generalizable to other populations. Lastly, certain other factors that may influence susceptibility to HCV infection, such as socioeconomic status and behavioral risk factors, were not examined in this study.

## Conclusions

Based on our findings, DAAS therapy is effective in the treatment of HCV patients. However, further research and real-world evidence are needed to deepen our understanding of DAAS therapy's long-term efficacy and safety in the Saudi population. These insights will help inform clinical guidelines and promote evidence-based decision-making for the management of HCV in Saudi Arabia. Also, these results contribute to the understanding of HCV infection epidemiology and clinical aspects, which can inform future research to further guide the development of targeted HCV interventions and treatment strategies. It should be borne in mind that this paper is based on secondary data. Additionally, the findings of this study may not be generalizable to other populations given the genetic variability of HCV. Hence, taking steps to improve the MOH surveillance data recording will help future researchers to study DAAS clinical safety among HCV patients in a more comprehensive manner.
